# STORM Super-Resolution Visualization of Self-Assembled γPFD Chaperone Ultrastructures in *Methanocaldococcus jannaschii*

**DOI:** 10.1021/acs.nanolett.4c01043

**Published:** 2024-05-09

**Authors:** Hee-Jeong Cha, Changdong He, Dominic J. Glover, Ke Xu, Douglas S. Clark

**Affiliations:** † Department of Chemical and Biomolecular Engineering, University of California—Berkeley, Berkeley, California 94720, United States; ‡ Department of Chemistry, University of California—Berkeley, Berkeley, California 94720, United States; § School of Biotechnology and Biomolecular Sciences, University of New South Wales, Sydney, NSW 2052, Australia; ∥ Molecular Biophysics and Integrated Bioimaging Division, Lawrence Berkeley National Laboratory, 1 Cyclotron Road, Berkeley, California 94720, United States

**Keywords:** archaeal chaperone, prefoldin, super-resolution microscopy, self-assembled nanostructures

## Abstract

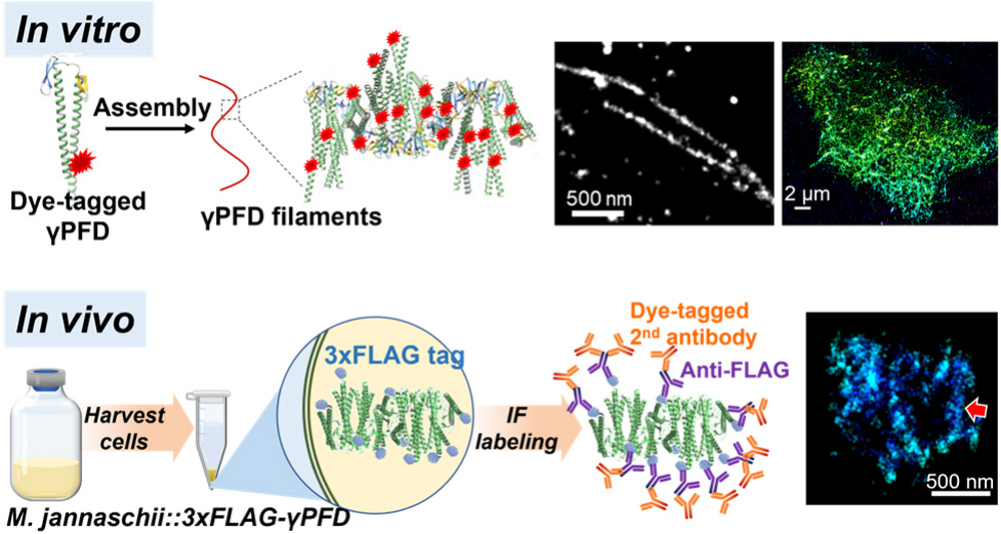

Gamma-prefoldin (γPFD), a unique chaperone found in the extremely thermophilic methanogen *Methanocaldococcus jannaschii*, self-assembles into filaments *in vitro*, which so far have been observed using transmission electron microscopy and cryo-electron microscopy. Utilizing three-dimensional stochastic optical reconstruction microscopy (3D-STORM), here we achieve ∼20 nm resolution by precisely locating individual fluorescent molecules, hence resolving γPFD ultrastructure both *in vitro* and *in vivo*. Through CF647 NHS ester labeling, we first demonstrate the accurate visualization of filaments and bundles with purified γPFD. Next, by implementing immunofluorescence labeling after creating a 3xFLAG-tagged γPFD strain, we successfully visualize γPFD in *M*. *jannaschii* cells. Through 3D-STORM and two-color STORM imaging with DNA, we show the widespread distribution of filamentous γPFD structures within the cell. These findings provide valuable insights into the structure and localization of γPFD, opening up possibilities for studying intriguing nanoscale components not only in archaea but also in other microorganisms.

Molecular chaperones are essential for nascent protein chains to properly fold after translation and play an important role in protein quality control in cells. They recognize hydrophobic sequences and can hand off partially folded polypeptides to chaperonins to facilitate proper folding, or transfer noncompetent proteins to the cell’s degradation machinery.^1,2^ Prefoldin (PFD) binds to misfolded proteins and transports them to Group II chaperonins (for example, the thermosome in archaea or CCT complexes in eukaryotes), which assist in the downstream folding of proteins into their functional conformations.^3^ The tentacle-like coiled-coil structure interacts with misfolded proteins through hydrophobic interactions and enables the transfer of the misfolded protein to a chaperonin.^3−5^

*Methanocaldococcus jannaschii*, a hyperthermophilic archaeon discovered near a deep-sea hydrothermal vent,^6^ exhibits a 26-fold increase in the transcription level of gamma-prefoldin (γPFD) in response to lethal heat shock treatments.^7^ γPFD is encoded by a homologous gene of αPFD but does not interact with either the α or β subunit. γPFD is a 16-kDa monomer and has the unique feature of forming self-assembled filaments *in vitro* through interactions between its β-sheet domains; their filamentous coiled-coil structure was recently confirmed by cryo-EM.^8^ The *in vitro* filaments are ∼6–8 nm in width, and exhibit varying lengths, extending to >2 μm.^9,10^ However, it remains unclear what structural form γPFD assumes within *M. jannaschii*, where it localizes, or what functional role(s) it plays in the host cell.

Previous imaging of archaeal structures has often employed electron microscopy. Earlier studies focusing on the archaeal envelope structure, including the S-layer and flagella, have relied primarily on scanning electron microscopy (SEM). For *M. jannaschii*, the ruptured form of the cell envelope following decompression from high pressure has been imaged with SEM.^11^ Transmission electron microscopy (TEM) has also been used to observe the cell membranes and internal components of *M. jannaschii*, including the cell surface layer, cytoplasmic membrane, and ribosomes.^6,12^ However, electron microscopy lacks molecular specificity and cannot identify γPFD in the crowded cell.

While fluorescence microscopy provides the desired molecular specificity,^13−15^ the ∼1 μm size of archaea^16^ makes it difficult to resolve subcellular structures with the ∼300 nm resolution afforded by conventional, diffraction-limited microscopy. Recent advances in super-resolution microscopy (SRM) have enabled subdiffractional imaging in biological specimens^17,18^ and *in vitro* filaments.^19−23^ For archaeal imaging,^15^ 3D-structured illumination microscopy, super-resolution radial fluctuations, stimulated emission depletion microscopy, and fluorescence photoactivated localization microscopy have been applied to investigate DNA replication,^24^ cell division,^25−28^ and spatial organization,^29^ bringing valuable sights down to ∼100 nm spatial resolution.

Here we report the application of single-molecule localization microscopy (SMLM),^30−32^ in particular three-dimensional stochastic optical reconstruction microscopy (3D-STORM),^33,34^ to elucidate the spatial organization of γPFD in *M. jannaschii* cells at ∼20 nm resolution. Starting with samples assembled *in vitro*, we first establish 3D-STORM as a suitable tool to examine γPFD nanostructures, resolving individual filaments, nanoclusters, and entangled bundles commensurate with TEM results. By next applying 3D-STORM to immunolabeled γPFD in *M. jannaschii*, we then demonstrate the existence of a filamentous form of γPFD, and further note their extensive, homogeneous distribution inside the cells, versus the compact arrangement of DNA at the cell center. Beyond unveiling the intriguing filamentous structure of γPFD in the *M. jannaschii* cell, our demonstrated application of 3D-STORM SRM also opens new paths for understanding archaea at the nanoscale.

We start by demonstrating STORM for γPFD filaments assembled *in vitro*. Purified His-tag γPFD monomers were labeled (Figure 1A) with the CF647 dye through NHS ester chemistry in a buffer containing 8 M guanidinium–HCl, a condition designed to inhibit γPFD self-assembly. Subsequently, filament incubation was initiated by buffer-exchanging to a 20 mM NaH_2_PO_4_, 150 mM NaCl solution and agitating it at 40 °C for 18 h. A substantial increase in the solution’s turbidity indicated successful filament formation. TEM of the 40 mM HEPES buffer-diluted suspension showed well-formed filaments (Figure 1B).

**Figure 1:**
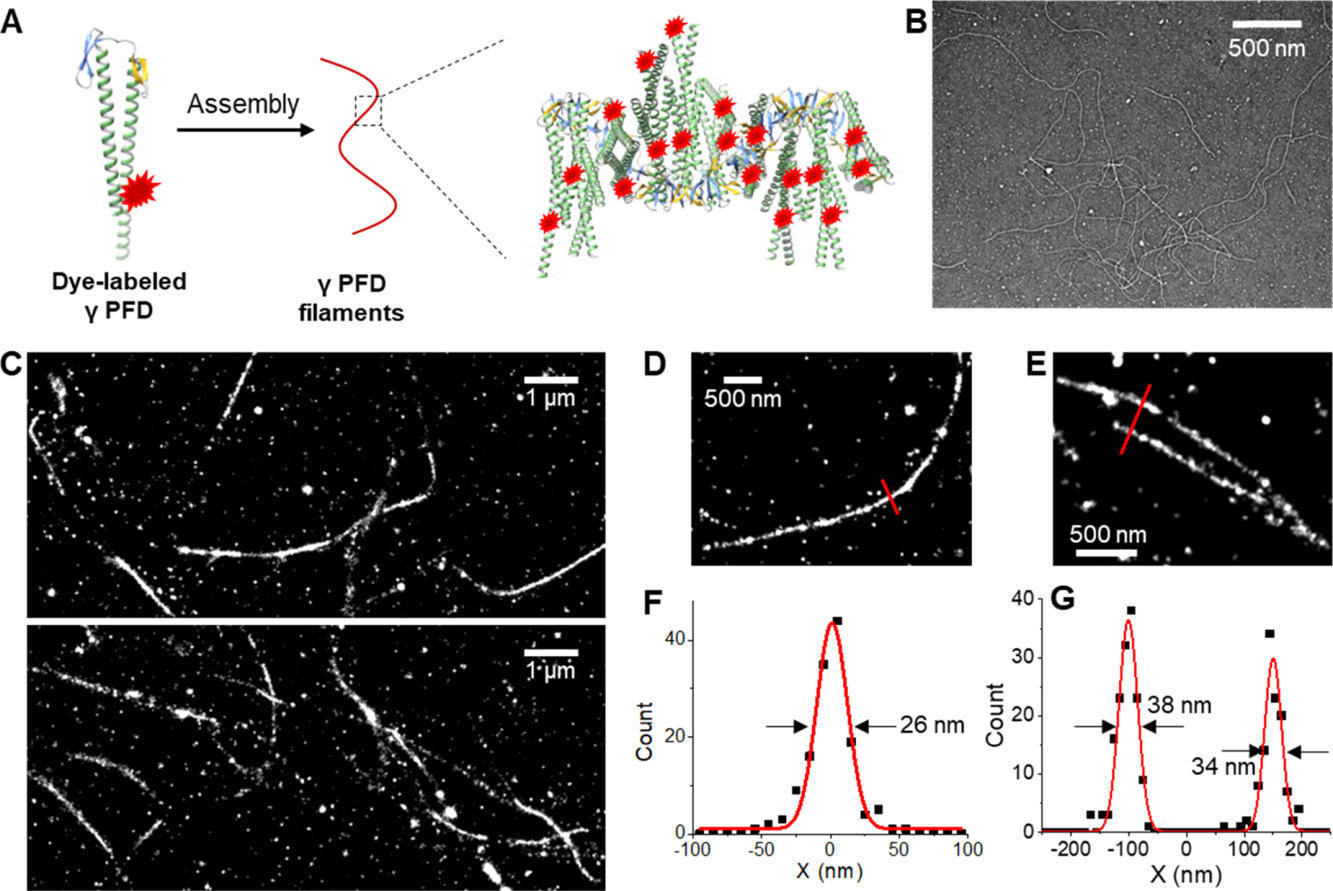
STORM characterization of assembled γPFD filaments *in vitro*. (A) Scheme: *in vitro* self-assembly of dye-labeled γPFD. (B) TEM of the *in vitro* assembled, dye-labeled γPFD filaments. (C–E) STORM images of the *in vitro* assembled γPFD. (F–G) STORM intensity profiles along the red lines in (D) and (E) (dots: experimental data, lines: fits with Gaussian peaks).

The diluted suspension was also dropped onto a coverslip for drying overnight at room temperature. The sample was then mounted with an oxygen-removed imaging buffer containing 2-aminoethanethiol, which facilitated the photoswitching of CF647 dyes.^33,34^ Under standard STORM imaging conditions, the labeled CF647 stochastically photoswitched between emitting and dark states, thus leaving a sparse set of single molecules emitting in the wide field for any given frame. Super-localizing the resultant blinking single-molecule images collected over ∼10^4^ frames thus enabled the reconstruction of super-resolution images at ∼20 nm resolution.

The resultant STORM images (Figure 1C-E) showed well-resolved individual filaments up to ∼5 μm in length, as well as spotty nanoclusters. These observations are in general agreement with the TEM results showing commensurate γPFD filament and oligomer structures (Figure 1B). STORM intensity profiles across individual filaments displayed a narrow full width at half maximum (FWHM) of ∼30 nm (Figure 1F, 1G and Figure S1), consistent with the ∼20 nm spatial resolution of STORM convolved with the γPFD filament width (8 nm). Figure 1E, 1G further demonstrate a case in which two filaments were well resolved at an ∼250 nm center-to-center distance below the diffraction limit of conventional fluorescence microscopy.

In addition to individual filaments, we also noticed high-density networks of filaments likely caused by fiber cross-interaction. To further elucidate these dense structures, inspired by our previous study on dense actin filaments in mammalian cells,^35^ 3D-STORM was employed to differentiate filaments at different sample depths. Figure 2A presents the full 3D structure of the dense filaments in which different colors represented the depth *Z*. By segregating the 3D-STORM data into 250 nm-thick virtual sections based on *Z*, we uncovered entangled filaments running in different directions at different depths, as well as isolated nanoclusters at the coverslip surface with the lowest *Z* values (Figure 2B-D). Zoom-in of the entangled γPFD bundles (Figure 2E) showed larger FWHM values of ∼90 nm (Figure 2F) versus the ∼30 nm values observed above for single filaments. TEM data also occasionally noted bundles significantly thicker than single filaments (arrows in Figure 2G).

**Figure 2:**
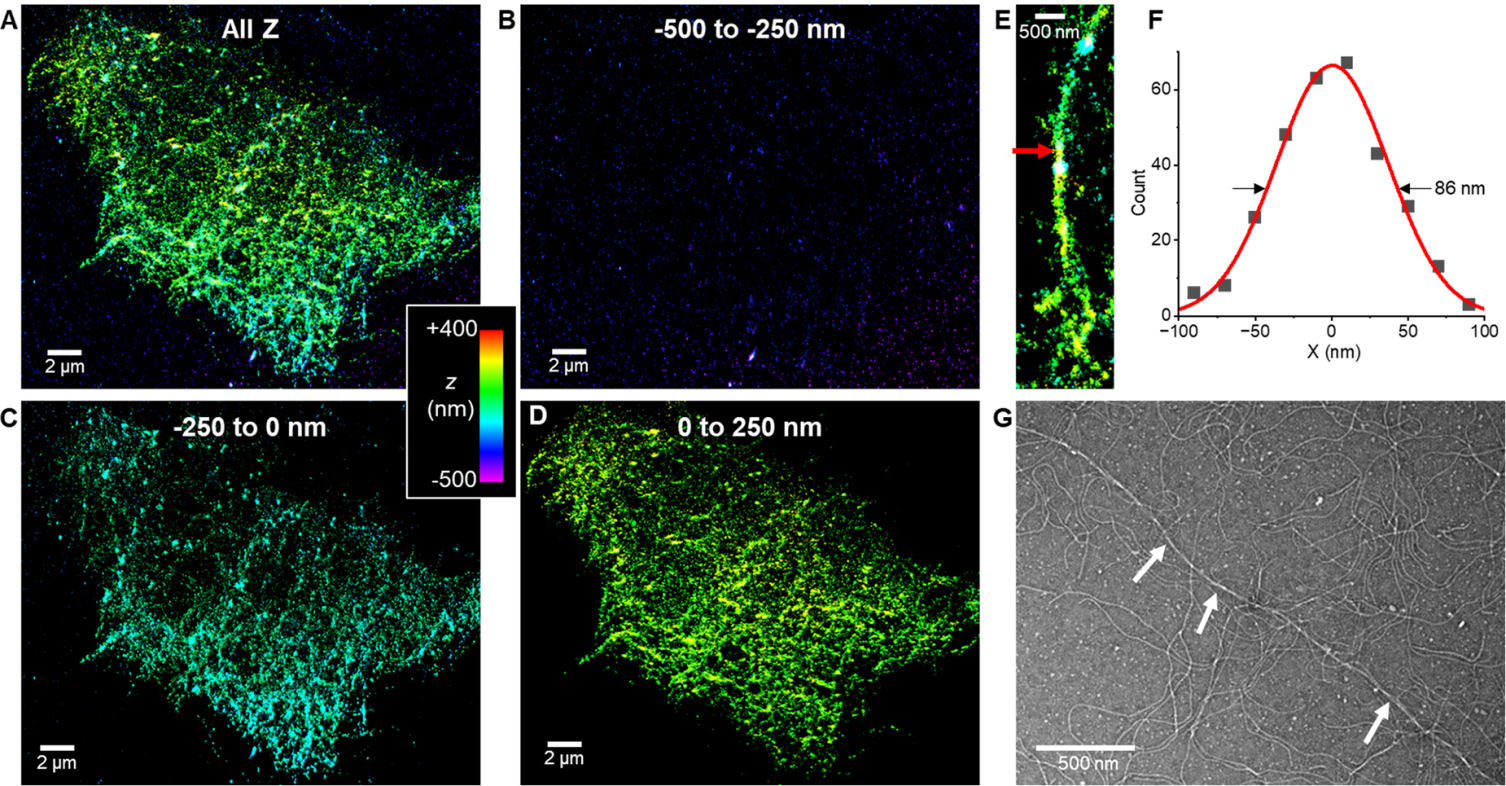
3D-STORM characterization of entangled γPFD filaments. (A) 3D-STORM image of entangled γPFD filaments, with the depth *Z* encoded as color (color scale bar). (B–D) Separation of (A) by *Z* for (B) −500 to −250 nm (C) −250 to 0 nm, and (D) 0 to 250 nm, with *Z* = 0 being the center of the imaging focal plane. (E) Example close-up 3D-STORM image of an entangled γPFD filament bundle. (F) STORM intensity profile at the position highlighted by the arrow in (E) (dots: experimental data, line: Gaussian fit). (G) TEM image showing entangled γPFD. Arrows point to a bundle notably thicker than filaments in the same view.

We also compared results based on a different labeling strategy. Unlabeled His-tag γPFD was assembled into filaments, deposited onto the coverslip, and then labeled through indirect immunofluorescence using an anti-His-tag primary antibody and a secondary antibody tagged by Alexa Fluor 647 (AF647). STORM similarly well-resolved single filaments (Figure S2), but yielded slightly wider widths of ∼40 nm FWHM versus the ∼30 nm width of filaments assembled from dye-labeled γPFD, attributable to the antibody sizes.

With the above successful demonstration of 3D-STORM for resolving single γPFD filaments and bundles *in vitro*, we proceeded to image γPFD in the *M. jannaschii* host cell. To this end, we expressed 3×FLAG-γPFD in *M. jannaschii* so that immunofluorescence could be achieved using an anti-FLAG tag antibody. To confirm that 3×FLAG-γPFD can correctly integrate into γPFD filaments, we separately purified 3×FLAG-γPFD and show that its *in vitro* mixtures with His-tag γPFD at varying mole fractions of 0%, 25%, 50%, 75%, and 100% all generated undifferentiable filaments as observed by TEM (Figure S3).

Consequently, we constructed a plasmid encoding γPFD with a 3×FLAG tag attached to the N-terminus using the primers (Table S1 and Table S2), transformed it into *M. jannaschii*, and expressed the 3×FLAG-γPFD protein. The plasmid was integrated into the chromosomal DNA through a double cross-over homologous recombination (Figure S4). To confirm the identity of the transformants, we performed PCR and sequencing to assess the size and sequence of the integrated gene (Figure S5A). Additionally, RT-qPCR was employed for the comparison of transcription level (Figure S5B). The *M. jannaschii* 3×FLAG-γPFD transformants were anaerobically cultured in medium 1. After 18 h, the cells were harvested, fixed with paraformaldehyde, and labeled through indirect immunofluorescence using an anti-FLAG tag primary antibody and a secondary antibody tagged by AF647 (Figure 3A). Once labeled, the cells were suspended in the STORM imaging buffer and deposited onto an agarose pad.^36^ A coverslip was delicately placed and secured on top of the sample. The sandwich setup was subsequently placed on the STORM microscope for imaging.

**Figure 3:**
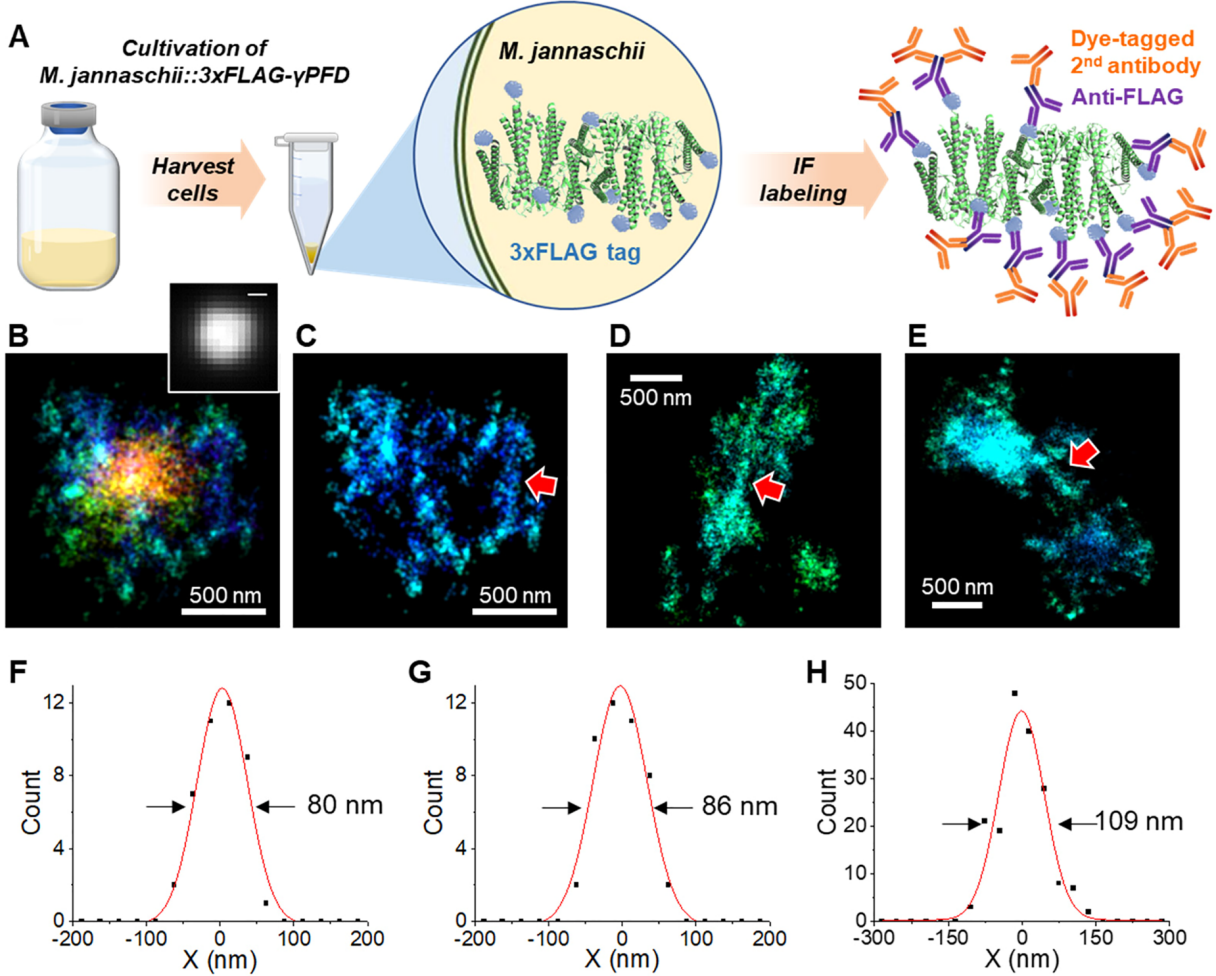
3D-STORM of γPFD in *M. jannaschii*. (A) Scheme of cell culturing and immunolabeling. (B) 3D-STORM image of an immunolabeled *M. jannaschii* cell (color-coded for depth *Z* from −800 to +800 nm, with *Z* = 0 being the center of the focal plane). Figure insets: diffraction-limited microscopy of the same area of the sample. (C) A 400 nm thick Z-section of the data in (B), to visualize the filamentous structures at the center of the cell. (D, E) 3D-STORM images of two other cells, shown as 400 nm thick Z-sections to visualize filamentous structures in the cells. (F–H) STORM intensity profiles at the positions highlighted by the arrow in (C–E) (dots: experimental data, lines: Gaussian fits).

Conventional diffraction-limited images of γPFD in the labeled cells showed a homogeneous signal (Figure 3B inset). In contrast, 3D-STORM resolved intricate details of γPFD inside the ∼1 μm-sized cells (Figure 3B and Figure S6). Using the same strategy above for filament networks, we divided the 3D-STORM signal into different virtual *Z* sections. Filamentous structures up to ∼500 nm long were thus observed as we examined the 400-nm-thick middle sections of different cells (Figure 3C-E). The STORM intensity profile of the structures displayed FWHM ∼70–120 nm (Figure 3F–H and Figure S7), larger than the above-observed FWHM of *in vitro* single filaments, yet comparable to the *in vitro* bundle structures. The filamentous structures were distributed relatively homogeneously inside the cells, with some oriented along the elongated cell shapes (Figure 3D, 3E). We also compared *M. jannaschii* expressing His-tag γPFD fixed with the stronger fixative of a paraformaldehyde–glutaraldehyde mixture, and observed comparable 3D-STORM results (Figure S8).

To confirm that the diverse cell shapes we observed with the 3D-STORM of γPFD are native to *M. jannaschii*, we further imaged the membrane of live cells using 10 nM BDP-TMR, a dye that enables SMLM through the reversible binding to lipid membranes.^37,38^ We thus visualized both round and elongated cell shapes (Figure S9), consistent with the γPFD filament-filled space observed above.

To further understand the distribution of γPFD in the cell, we next performed two-color STORM for γPFD and the DNA, with the latter probed by the dye NucSpot Live 488. Sequential STORM imaging in two color channels thus indicated that DNA was concentrated at the center of the cells as compact, elongated shapes, whereas the γPFD filaments were distributed more extensively in the cell (Figure 4). As a control, two-color STORM of similarly stained wild-type *M. jannaschii* cells with no expression of 3×FLAG-γPFD showed comparable DNA structures but no labeling in the AF647 channel (Figure S10).

**Figure 4:**
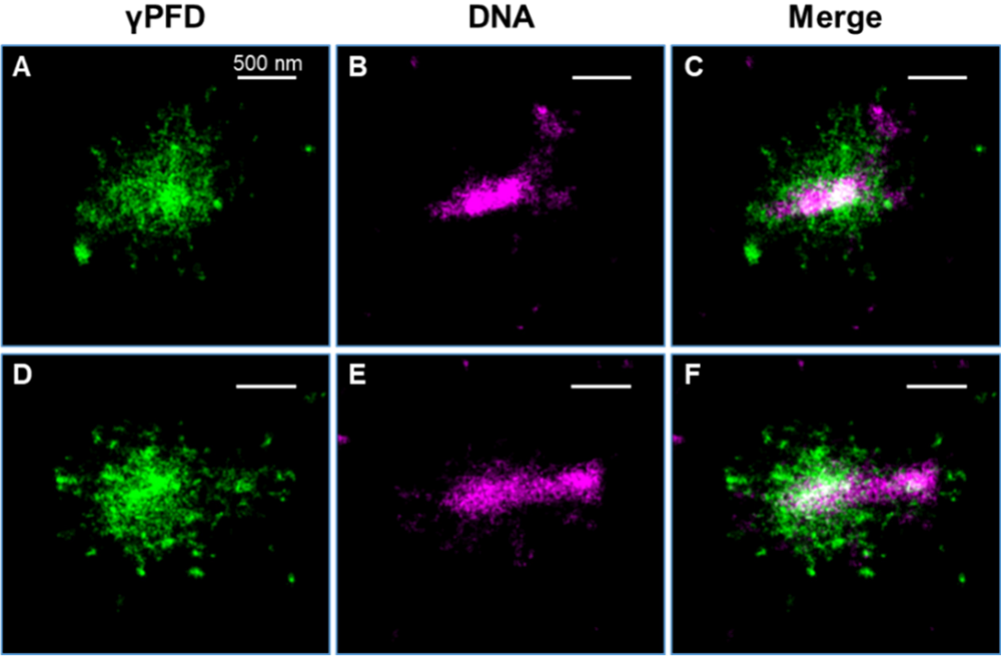
Two-color STORM imaging of γPFD filaments (AF647) and DNA (NucSpot Live 488) in *M. jannaschii*. (A and D) STORM of γPFD filaments was first performed in the 647 channel. (B and E) PAINT of DNA in the 488 channel. (C and F) Overlaid images of the 647 and 488 channels.

In conclusion, this study utilized SMLM, in particular 3D-STORM, to gain insights into the spatial organization of γPFD both *in vitro* and *in vivo*. Our 3D-STORM imaging validated the presence of filamentous γPFD structures within crowded cells, an observation that exceeds the capabilities of electron microscopy. Furthermore, by overcoming the diffraction limitations of fluorescence microscopy, STORM imaging allowed us to achieve nanoscale observations of γPFD in archaea.

The study initially focused on successfully using STORM to visualize individual γPFD filaments and nanoclusters *in vitro*. We were able to reproduce the filamentous structure of γPFD, as observed in transmission electron microscopy (TEM) and cryo-electron microscopy (cryo-EM), by labeling with CF647 NHS ester in STORM. The observed filament width in STORM was ∼30 nm compared to the established width of 8 nm, consistent with ∼20 nm optical resolution convolved with the actual filament width. Additionally, our investigation confirmed that γPFD can form bundles of entangled filaments with FWHM values of ∼90 nm.

By expressing 3×FLAG-tagged γPFD and utilizing immunofluorescence, 3D-STORM imaging was employed to visualize the distribution of γPFD inside *M. jannaschii* cells. This revealed that γPFD is uniformly distributed within the cells and often follows the elongated shapes of the cells. Two-color STORM imaging of γPFD and DNA demonstrated that while DNA is concentrated at the center of the cells in a compact manner, γPFD surrounds the DNA and extends throughout the cell. Although this study did not establish conclusive evidence of γPFD–DNA interactions, it highlights the potential for future investigations to explore protein–protein and protein–DNA interactions using this imaging technique. Together, beyond unveiling the intriguing filamentous structure of γPFD in *M. jannaschii*, our results open new possibilities for understanding the nanoscale intracellular localization of specific proteins in archaea.

## Supporting Information Available

Materials and Methods; Description and characterization of plasmids and primers used in this work; Additional quantification of filament widths; *in vitro* and *in vivo* STORM results of His-tag γPFD based on anti-His tag immunofluorescence; STORM of live *M. jannaschii* membrane and control samples (PDF)
