# Supplementary material for: STORM Super-Resolution Visualization of Self-Assembled γPFD Chaperone Ultrastructures in Methanocaldococcus jannaschii
Source: Nano Lett. 2024 May 9;24(20):6078–83. doi: 10.1021/acs.nanolett.4c01043 (PMC11117396; doi:10.1021/acs.nanolett.4c01043)
Supplement: Supplementary file 1 — nl4c01043_si_001.pdf [file nl4c01043_si_001.pdf]

Supporting Information

STORM super-resolution visualization of self-assembled γPFD chaperone ultrastructures in *Methanocaldococcus jannaschii*

Hee-Jeong Cha[||,1], Changdong He[||,2], Dominic J. Glover[3], Ke Xu*[2,4], Douglas S. Clark*[1,4]

[||]Co-first authors

*Corresponding authors. xuk@berkeley.edu, dsc@berkeley.edu

[1]Department of Chemical and Biomolecular Engineering, University of California, Berkeley, Berkeley, CA 94720, USA

[2]Department of Chemistry, University of California, Berkeley, Berkeley, CA 94720, USA

[3]School of Biotechnology and Biomolecular Sciences, University of New South Wales, Sydney, NSW 2052, Australia

[4]Molecular Biophysics and Integrated Bioimaging Division, Lawrence Berkeley National Laboratory, 1 Cyclotron Road, Berkeley, CA 94720, USA

MATERIALS & METHODS

Materials

Mevinolin (Lovastatin), cysteine, and $Na_2S \cdot 9H_2O$ were purchased from Sigma (St. Louis, MO, USA). All restriction enzymes and Monarch chromosomal DNA prep kit were obtained from New England Biolabs (NEB). In-Fusion cloning kit was from Takara (Takara Bio, Inc., Mountain View, CA). CF647-NHS ester and NucSpot 488 were from Biotium. Mouse monoclonal anti-6x-His Tag (HHHHHH) was from Thermo Fisher (#MA1-21315). Rabbit monoclonal anti-FLAG Tag (DYKDDDDK) was from Cell Signaling Technology (#14793). Alexa Fluor 647-conjugated goat anti-rabbit IgG and goat anti-mouse IgG were from Thermo Fisher (A-21245 and A-21235). BDP-TMR-alkyne was from Lumiprobe (A24B0).

Purification of His-tag γPFD and 3x-FLAG γPFD

γPFD was expressed and purified as described previously.[1, 2] His-tag (6x-His) γPFD and 3x-FLAG γPFD encoded plasmids were separately transformed into BL21 T7 Express *Escherichia coli* (NEB) and cultured in LB medium containing 100 μg mL^{-1} ampicillin at 37°C, 250 rpm, until the optical density (OD) reached 0.6. The expression of tagged γPFD was induced with a final concentration of 1 mM isopropyl β-D-1-thiogalactopyranoside (IPTG). Transformed cells were incubated at 25°C, 200 rpm for 18 h, then centrifuged at 6000 x g for 20 min, resuspended in a 50 mM NaH_2PO_4, 100 mM NaCl buffer, pH 8.0, and lysed using sonication. The lysate was then filtered through a 0.22-μm filter. The 6x-His γPFD was purified using Ni-NTA agarose (Qiagen), washed twice with 20 mM imidazole, 50 mM NaH_2PO_4, and 300 mM NaCl, pH 8.0 buffer. The 6x-His γPFD was then eluted using 250 mM imidazole, 50 mM NaH_2PO_4 buffer, 300 mM NaCl, pH 8.0. 3x-FLAG γPFD was purified by multimodal chromatography (MMC), as described previously.[1, 2] Briefly, Capto Core 700 resin (Cytiva) was packed into polypropylene columns (Qiagen), and the effluent was collected by gravity flow, with fraction volumes of 1 mL. To each fraction was added 10 μL Triton X-114, followed by incubation at 37°C for 5 min and centrifugation at 13000 rpm for 1 min to facilitate phase partitioning. The protein concentration in the supernatant was determined using the BCA assay.

Dye labeling of purified γPFD

γPFD monomer was labeled by mixing CF647-NHS ester and purified His-tag γPFD (~0.6 mg/mL) at a molar ratio of 4:1 in an 8 M guanidinium–HCl, 10 mM NaH_2PO_4, pH 8.0 buffer and incubating at room temperature overnight. Excess dye was removed using Amicon 3k MWCO Centrifugal Filters (Millipore, UFC500396). The dye and protein concentrations are determined by measuring absorbances at 650 nm with a NanoDrop 2000c spectrometer (ThermoFisher) and Pierce™ Rapid Gold BCA Protein Assay Kit (ThermoFisher A53225). The final dye-to-protein ratio was ~1:1.

In vitro assembly of purified γPFD

For the *in vitro* assembly of dye-labeled γPFD, the dye-labeled γPFD was buffer-exchanged to the refolding buffer (20 mM NaH_2PO_4, 150 mM NaCl, pH 8.0) using Amicon 3k MWCO Centrifugal Filters, and the sample was incubated at 40°C for 18 h. For the assembly of unlabeled γPFD, the purified His-tag γPFD and 3x-FLAG γPFD were mixed in 8 M guanidine-HCl at different mole ratios of 0%, 25%, 50%, 75%, and 100% of 3x-FLAG γPFD, and then dialyzed against the PBS buffer overnight using a 3.5K Slide-A-Lyzer™ mini dialysis cup.

TEM imaging of *in vitro* filaments

TEM was performed as described previously.[1, 2] The assembled proteins were diluted to 0.12 µM in 40 mM HEPES buffer, pH 7.4. The sample was placed on 400-mesh carbon/formvar-coated copper grids (EMS) and stained with 1% uranyl acetate. TEM imaging was conducted using a FEI Tecnai 12 transmission electron microscope operating at 120 kV.

Organisms and culture conditions

Methanocaldococcus jannaschii DSM2661 was purchased from Leibniz Institute DSMZ-German Collection of Microorganisms and Cell Cultures GmbH. *M. jannaschii* was cultivated as previously reported.[3] Briefly, *M. jannaschii* were grown in the medium1 using Hungate tubes or 160-ml serum bottles with sealed butyl rubber stoppers and aluminum crimp caps under strict anaerobic conditions. Medium1 comprised the following components and concentrations (g/L): K_2HPO_4, 0.055; KH_2PO_4, 0.055; KCl, 1; NaCl, 25; $NaHCO_3$, 0.84; $CaCl_2{\cdot}2H_2O$, 0.36; $MgCl_2{\cdot}6H_2O$, 7.7; NH_4Cl, 1.18; $Fe(NH_4)_2(SO_4)_2{\cdot}6H_2O$, 0.01; Na_3-nitrilotriacetate, 0.082; Na_2SeO_4, 0.0003; $Na_2WO_4{\cdot}2H_2O$, 0.003; $Na_2MoO_4{\cdot}2H_2O$, 0.002; yeast extract and tryptone 2. A 100-fold-concentrated trace mineral solution was added to the medium1 (10 ml/L) to achieve the final concentrations (µM): nitrilotriacetic acid, 71; $MnCl_2{\cdot}4H_2O$, 4.5; $FeCl_2{\cdot}4H_2O$, 6.8; $CaCl_2$, 4.1; $CoCl_2{\cdot}6H_2O$, 7.6; $ZnCl_2$, 6.6; $CuSO_4$, 2.8; $Na_2{\cdot}MoO_4{\cdot}2H_2O$, 1.9; $NiCl_2{\cdot}6H_2O$, 3.8, and the final pH was about 6.2. The medium1 was reduced by adding $Na_2S{\cdot}9H_2O$ solution and cysteine-HCl solution, each to a final concentration of 2 mM, and the final pH was adjusted to 6.0 with sterilized HCl. Fresh medium1 was inoculated with a 10% stock of *M. jannaschii* and pressurized to 30 psi with a mixture of H_2 and CO_2 (80:20, v/v). After selecting transformants, the cell stocks were serially subcultured every 3 weeks by transferring them to a fresh medium and kept at 4℃. The inoculum was incubated using a shaking water bath (Thermo Scientific Precision Water Baths, SWB 15, Pittsburgh, PA) at 85℃ and 200 rpm.

Gene cloning and construction of plasmids

To manipulate the chromosomal DNA of *M. jannaschii*, suicide plasmids were constructed (Table S1) and integrated into chromosomal DNA by double cross-over homologous recombination (Figure S4), as described by Susanti and coworkers.[3] The designed primers are presented in Table S2. *M. jannaschii* genomic DNA was isolated using the Monarch genomic DNA purification kit (NEB). Each suicide plasmid contained the ~502 bp and ~504 bp

of the upstream and downstream gene of γPFD (locus tag, mj_0648) between *Not* Ⅰ and *Apa* Ⅰ restriction sites of the pDS210 vector as a template.[3] The DNA fragments were integrated using In-Fusion cloning kit.

Transformation of 3xFLAG-γPFD plasmid into *M. jannaschii*

To transform the plasmid, *M. jannaschii* was grown in medium1 at 60℃ to OD_{600nm} 0.4~0.5, which is the early exponential growth phase. The cells were resuspended in 0.4 ml of pre-reduced medium1 and supplemented with 2 µg of linearized plasmid by *Xmn* Ⅰ restriction enzyme. Subsequently, they were incubated at 4℃ for 1 h. The cells were then heat shocked at 85℃ for 45 sec and incubated at 4℃ for 10 min. Pre-reduced medium1 was then added to a volume of 5 ml, and the cells were cultivated at 80℃ in a water bath without shaking. After ~18 hr the cells were then spread onto Gelrite plates that contained 30 µM of the antibiotic Mevinolin. Afterwards, integration of the 3xFLAG tag into the chromosomal DNA of *M. jannaschii*::3xFLAG-γPFD (*Mj*::3xFLAG-γPFD) was confirmed via genotypic DNA characterization, involving PCR amplicon size comparison (Figure S5A) and sequencing (data not shown). Chromosomal DNA from *Mj*::3xFLAG-γPFD was isolated using the Monarch® Genomic DNA Purification Kit (NEB), and the integrated gene was amplified with primers through PCR (Figure S5A). Furthermore, transcription of the 3xFLAG-γPFD gene was established through RT-qPCR analysis (Figure S5B). RNA was extracted using TRIzol® reagent, followed by reverse transcription into cDNA using Superscript III (Invitrogen). qPCR was conducted on cDNA derived from the RT reaction using Power SYBR Green PCR Master mix (Applied Biosystems™), following the manufacturer's protocol. qPCR was carried out using a BioRad CFX Connect 96w (Bio-Rad, Hercules, CA).

Immunofluorescence labeling of 3xFLAG-γPFD and 6xHis-γPFD in *M. jannaschii*

The spacing between the adjacent dimer units in assembled γPFD filaments is known to be 2.3 nm, which can result in steric hindrance when large fusion proteins are used due to the dense attachment of the proteins along the filament.[4, 5] To overcome this limitation, we performed immunofluorescence labeling using a 3xFLAG-tag at the N-terminus of γPFD to enable visualization of the γPFD in whole cells. The cells were harvested by centrifuge 5,000 rpm for 5 min, and fixed by 4% paraformaldehyde for 15 min. The fixed cells were washed twice with CS (cytoskeleton) buffer (10 mM MES, 150 mM NaCl, 5 mM EGTA, 5 mM glucose, 5 mM $MgCl_2$, pH 6.1) containing 50 mM NH_4Cl, for 5 min with gentle shaking as following the previous study.[6-8] The cells were resuspended with CS buffer containing 5% bovine serum albumin (BSA) and 0.25% (v/v) Triton X-100. After 30 min of gentle shaking, primary antibody (Rabbit@FLAG) was added to the fixed cells at 1:200. The fixed cells were incubated with the primary antibody with gentle shaking for 2 h at 37°C and then overnight at 4°C. The cells were washed three times with CS (cytoskeleton) buffer containing 50 mM NH_4Cl, and then incubated for 2 h with secondary antibodies (Goat@Rabbit-AF647) diluted in CS buffer containing 5% BSA and 0.25% (v/v) Triton X-100. The sample was washed three times with CS buffer containing 50 mM NH_4Cl, and resuspended in DPBS. We note that

due to the extensive washing steps in our above labeling protocol for STORM, we ended up with low cell counts in the final suspension, thus limiting the number of cells to be recorded to just a few in the entire field of view for each STORM run (Figure S6), awaiting future optimizations for higher throughputs. In Figure S7, we further compared results with cells expressing 6x-His tagged γPFD. Cells were fixed with 3% paraformaldehyde plus 0.1% glutaraldehyde in DPBS for 15 min, and then washed twice with a freshly prepared 0.1% $NaBH_4$ solution in DPBS. The same immunolabeling procedure as above was next performed, but with Mouse@His-Tag as the primary antibody and Goat@Mouse-AF647 as the secondary antibody.

Preparation of samples for STORM imaging

In vitro filament samples were prepared by diluting the above filament stock solution with Milli-Q water at a ratio of 1 to 200. 50 μL of the solution was dropped onto a coverslip and dried at room temperature overnight. For His-tag γPFD not yet labeled by CF647, 20 μL of the filament stock was dropped onto a coverslip and incubated for 20 min followed by the addition of 200 μL of 5% BSA in DPBS. After 20 min incubation, 1 μL anti-His Tag antibody was directly diluted into the solution and then the sample was incubated overnight at 4°C. The attached filaments were washed twice with DPBS, and then incubated for 2 h with secondary antibodies (Goat@Mouse-AF647) diluted in DPBS containing 5% BSA. Excess secondary antibody was removed by washing twice with DPBS. The samples were then mounted onto a glass slide with a Tris-Cl imaging buffer (pH 7.5) containing 100 mM cysteamine, 5% glucose, 0.8 mg mL^{-1} glucose oxidase, and 40 μg mL^{-1} catalase. For the *in vivo* samples, immunofluorescence-labeled cells were suspended with the Tris-Cl imaging buffer described above and sandwiched between a gel pad[9] and a coverslip. For two color STORM of γPFD and DNA, the immunofluorescence-labeled cells were first labeled by NucSpot 488 (40081-T) for 20 min and washed twice by DPBS before suspending with the Tris-Cl imaging buffer. For live cell imaging, fresh cells are washed twice by DPBS and resuspended in 10 nM BDP-TMR in DPBS.

3D-STORM imaging

3D-STORM was performed on a homebuilt inverted microscope using a Nikon CFI Plan Apo λ 100x oil-immersion objective (NA = 1.45), as described previously.[10-12] Lasers of 647 nm (for CF647 and AF647) or 488 nm (for NucSpot 488) excited the sample at ~1.5 kW/cm^2. The angle of incidence was slightly below the critical angle of total internal reflection, thus illuminating a few micrometers into the sample. The relatively strong excitation powers switched most of the initially fluorescent molecules in the sample into a non-emitting dark state, leaving a small, random fraction of single molecules emitting in the view in any given frame. A 405-nm laser was applied to further assist the activation of the dark-state molecules back to the emitting state, with gradually increasing power of 0-5 W/cm^2 during the experiment, to sustain a suitable density of single molecules in the view, as described previously.[10] The resulting single-molecule fluorescence was recorded using an Andor iXon Ultra 897 EM-CCD at 110 frames per second. A total of ~30,000 frames and ~80,000 frames were recorded per image for *in vitro* and *in vivo* samples,

respectively. For 3D localization, a cylindrical lens was inserted to create astigmatism for encoding the single-molecule depth (*Z*) information.[13] The raw STORM data were analyzed using previously described methods.[13, 14]

Table S1. Suicide plasmid to construct *M. jannaschii* variants

Plasmid	Descriptions	Reference
**pDS210**	A suicide plasmid for the construction of *Δfsr* (locus tag, mj_0870) *M. jannaschii* strain	[3]
**3xFLAG-γPFD expression plasmid**	A suicide plasmid for the expression of 3xFLAG tag γPFD *M. jannaschii* strain expressed by *M. jannaschii* native promoter as wild-type γPFD level	This study

Table S2. Primers used in this study to construct the 3xFLAG-γPFD *in M. jannaschii*

Plasmid	Insert	Primers	Sequence
**3xFLAG-γPFD expression plasmid**	γPFD Upstream (502 bp)	Forward	5'- CCGCGGTGGCGGCCGCTTTATCTACACACTTTATTTTG -3'
		Reverse	5'- GAATATTTGGCGCGCCACTATCCCCTCTTTTATT -3'
	3xFLAG-γPFD	Forward	5'- GATAGTGGCGCGCCATGGACTATAAGG -3'
		Reverse	5'- GACTTCATTTACCATTTTATCATCGTCATCCTTATAATCAATATCATGATC -3'
	[γPFD, Psla-hmgA]	Forward	5'- CGATGATAAAATGGTAAATGAAGTCATAGACATAAATGAAGCAGTTAGAGC -3'
		Reverse	5'- AAACTCAGGCGCGCCTTATCTACCGAGTTC -3'
	γPFD Downstream (504 pb)	Forward	5'- AAGGCGCGCCTGAGTTTCAAGAAGTAATTGACT -3'
		Reverse	5'- CGAATTGGGTACCGGGCCCAGAAAAAAAAGCGTTGATG -3'

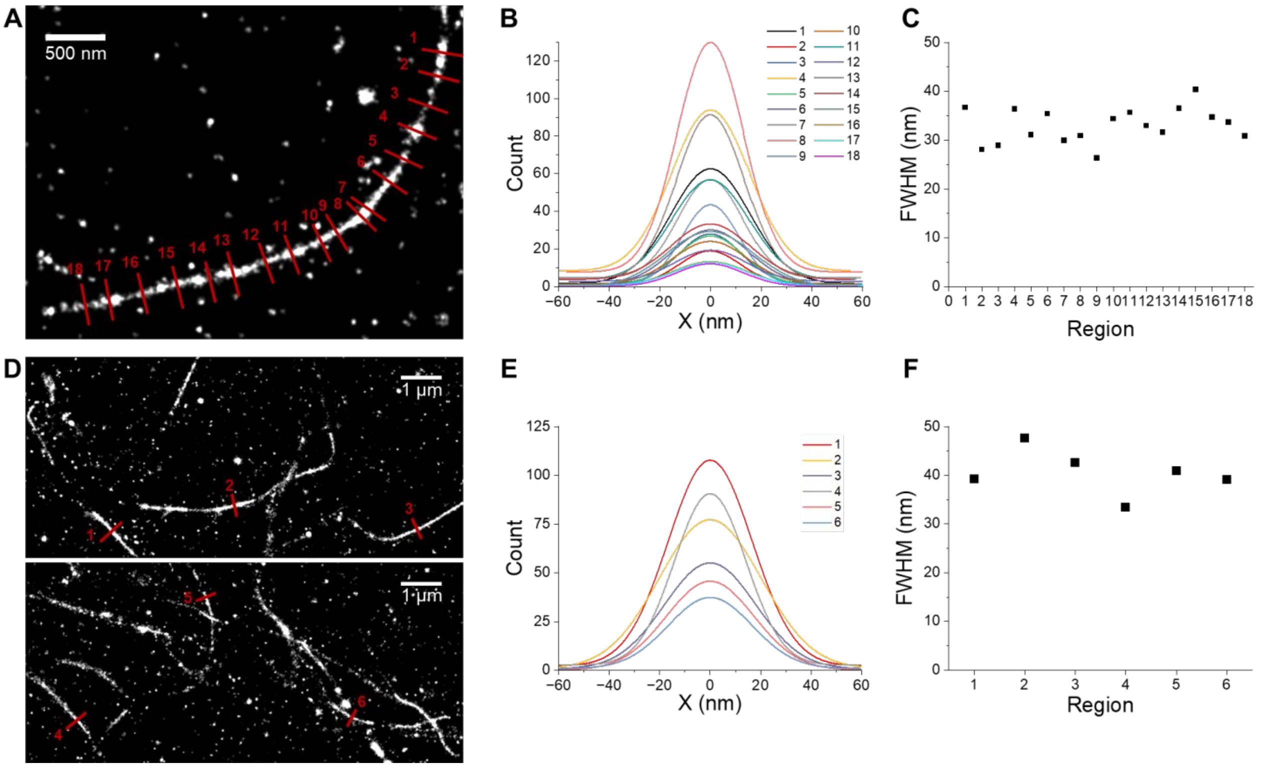

Figure S1. Quantification of the STORM-determined apparent filament widths for *in vitro* γPFD filaments assembled with dye-labeled γPFD. (**A**) Enlarged Figure 1D for the STORM images of one filament, with red lines marking where local cross sections are made. (**B**) STORM intensity profiles along the 18 red lines marked in (**A**), showing varying intensities but relatively invariant widths. (**C**) FWHM values of the different local cross sections. (**D**) STORM image of Figure 1C, with red lines marking local cross sections for different filaments. (**E**) STORM intensity profiles along the red lines across different filaments in (**D**). (**F**) Corresponding FWHM values of the cross sections of different filaments.

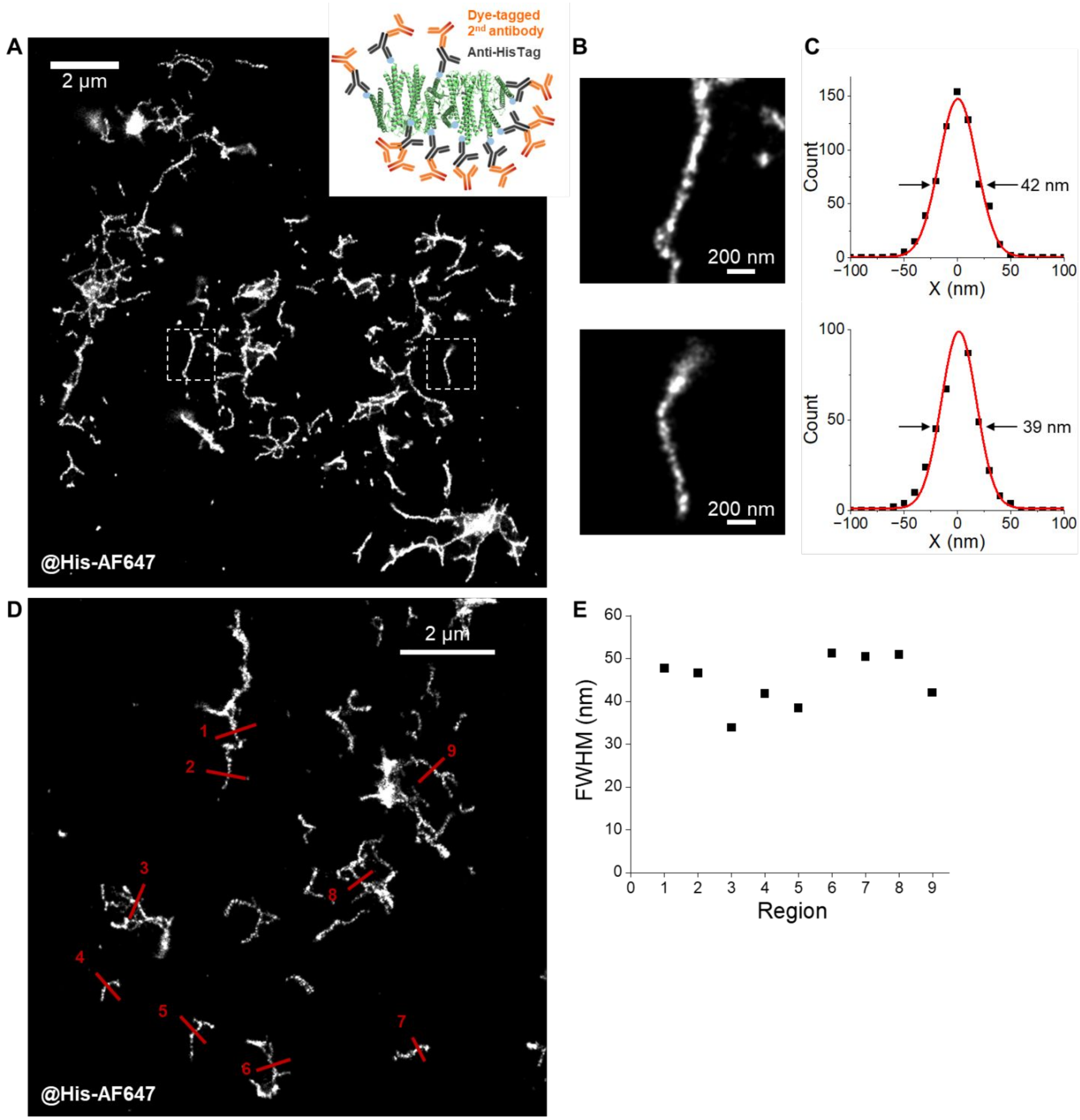

Figure S2. STORM of assembled γPFD filaments *in vitro* based on anti-His tag immunofluorescence labeling. (**A**) STORM image of a large view. Inset: labeling strategy. Unlabeled γPFD was first assembled into filaments, deposited on the coverslip, and then labeled using an anti-His-tag primary antibody and a secondary antibody tagged by Alexa Fluor 647 (AF647). (**B**) Zoom-in for the two boxed areas in (A), highlighting isolated single filaments. (**C**) STORM intensity across the centers of the two filaments, with FWHM marked in the plots. (**D**) STORM image of another sample. (**E**) FWHM values of cross sections of different filaments, along the red lines marked in (**D**).

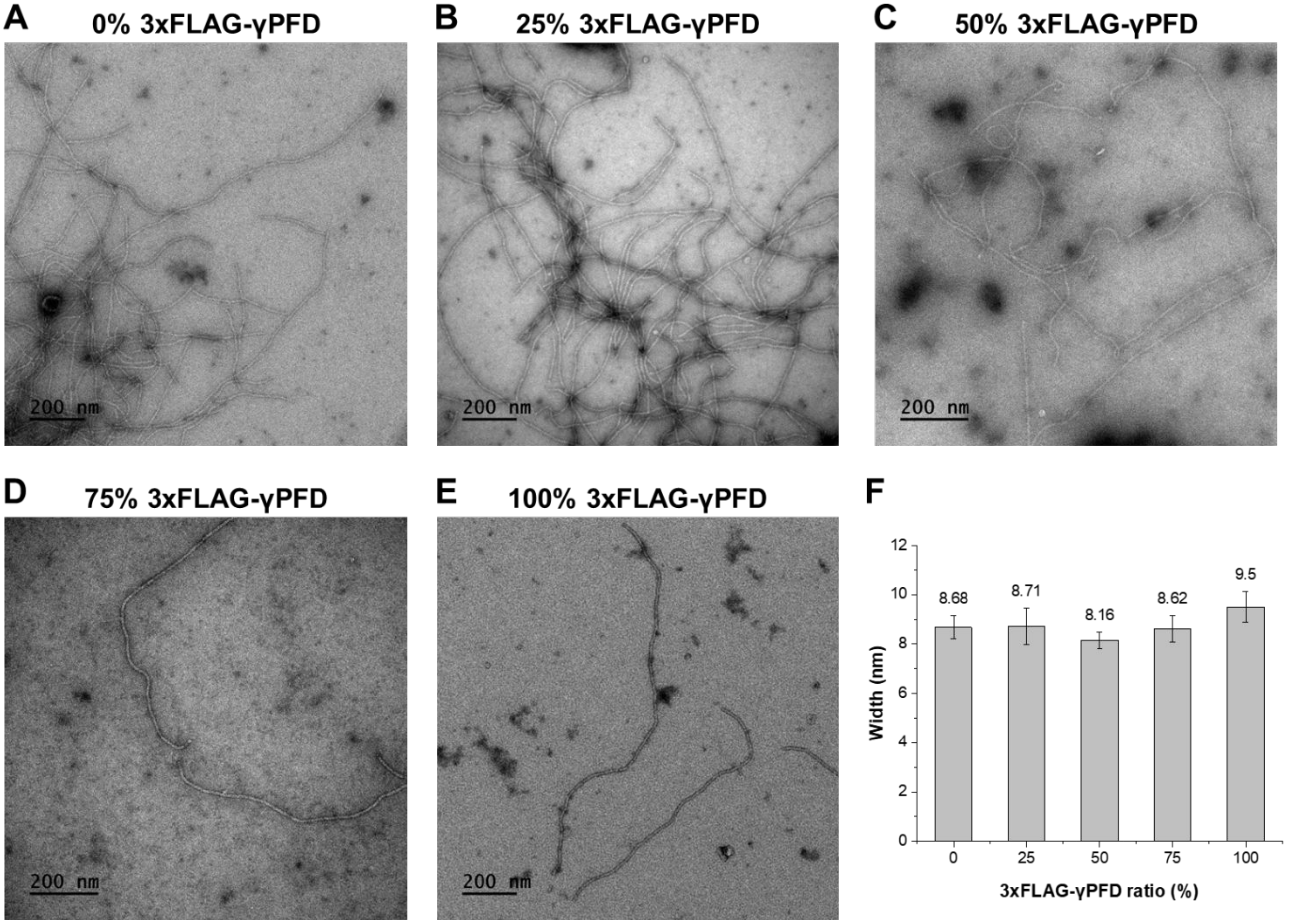

Figure S3. Comparison of TEM images of *in vitro* γPFD filaments assembled from varying mixtures of purified 3xFLAG-γPFD and His-tag γPFD. (**A**-**E**): Results of mixtures containing 0, 25%, 50%, 75%, and 100% 3xFLAG-γPFD. (**F**) Filament widths of the different samples.

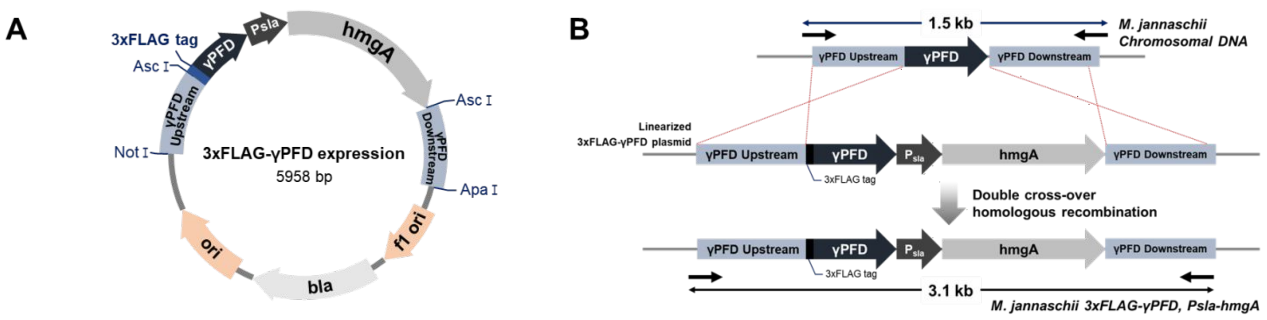

Figure S4. Suicide plasmid used to construct the 3xFLAG-γPFD strains. (**A**) 3xFLAG-γPFD expression plasmid. The region of γPFD upstream of 502 bp and downstream of 504 bp enables double cross-over homologous recombination in *M. jannaschii* cells. (**B**) *Mj*::3xFLAG-γPFD, Psla-hmgA strains were constructed by double cross-over recombination between chromosomal DNA of *M. jannaschii* and linearized 3xFLAG-γPFD plasmid including γPFD upstream (502 bp), 3xFLAG-γPFD (513 bp), Psla-hmgA (1,534 bp), and γPFD downstream (504 bp). The suicide plasmid included the specific *M. jannaschii* selectable gene cassette [Psla-hmgA], which includes the S-layer promoter of *Methanocaldococcus* FS.406-22, and encodes the HMG-CoA reductase gene of *M. jannaschii*. The hmgA gene imparts resistance to the antibiotic mevinolin. 3xFLAG-γPFD is expressed using the native promoter of *M. jannaschii*.

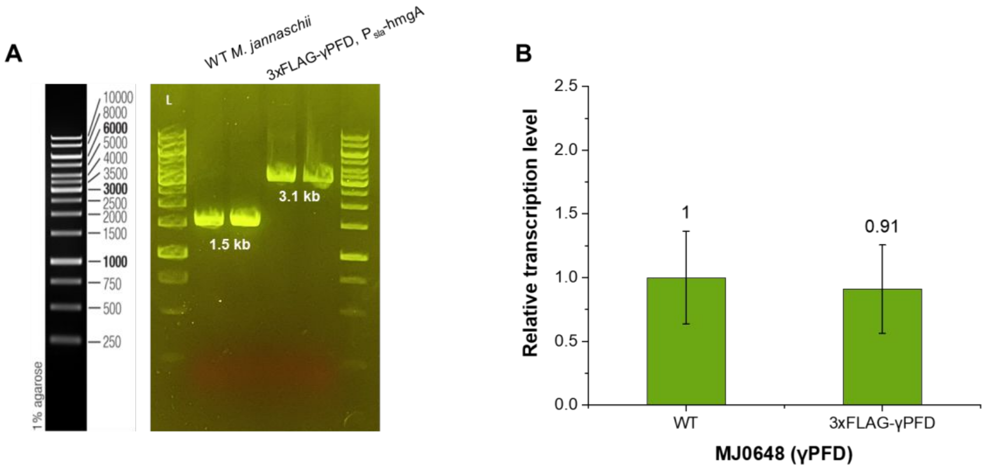

Figure S5. (**A**) Genotypic DNA characterization of *Mj*::3xFLAG-γPFD, Psla-hmgA strains using PCR. The primer F: 5'-GCTCTGCTCCAGTTGCTAAAACTAAG-3' and R: 5'-CCATTATGTTGAGGCGATGATGAAG-3' were used for the amplification of the integrated gene. The amplicon sizes were confirmed by electrophoresis on a 1% agarose gel. The wild-type amplicon size is 1.5 kb, and the *Mj*::3xFLAG-γPFD, Psla-hmgA amplicon size is 3.1 kb, as shown in Figure S4B. (**B**) Transcription level of mj_0648 (γPFD) in *Mj*::3xFLAG-γPFD, Psla-hmgA relative to the wild-type (WT) strain. Glyceraldehyde-3-phosphate dehydrogenase (GAPDH, mj_1411) was used as a reference gene. Relative transcription level and standard deviations were calculated based on two independent RT-qPCR experiments.

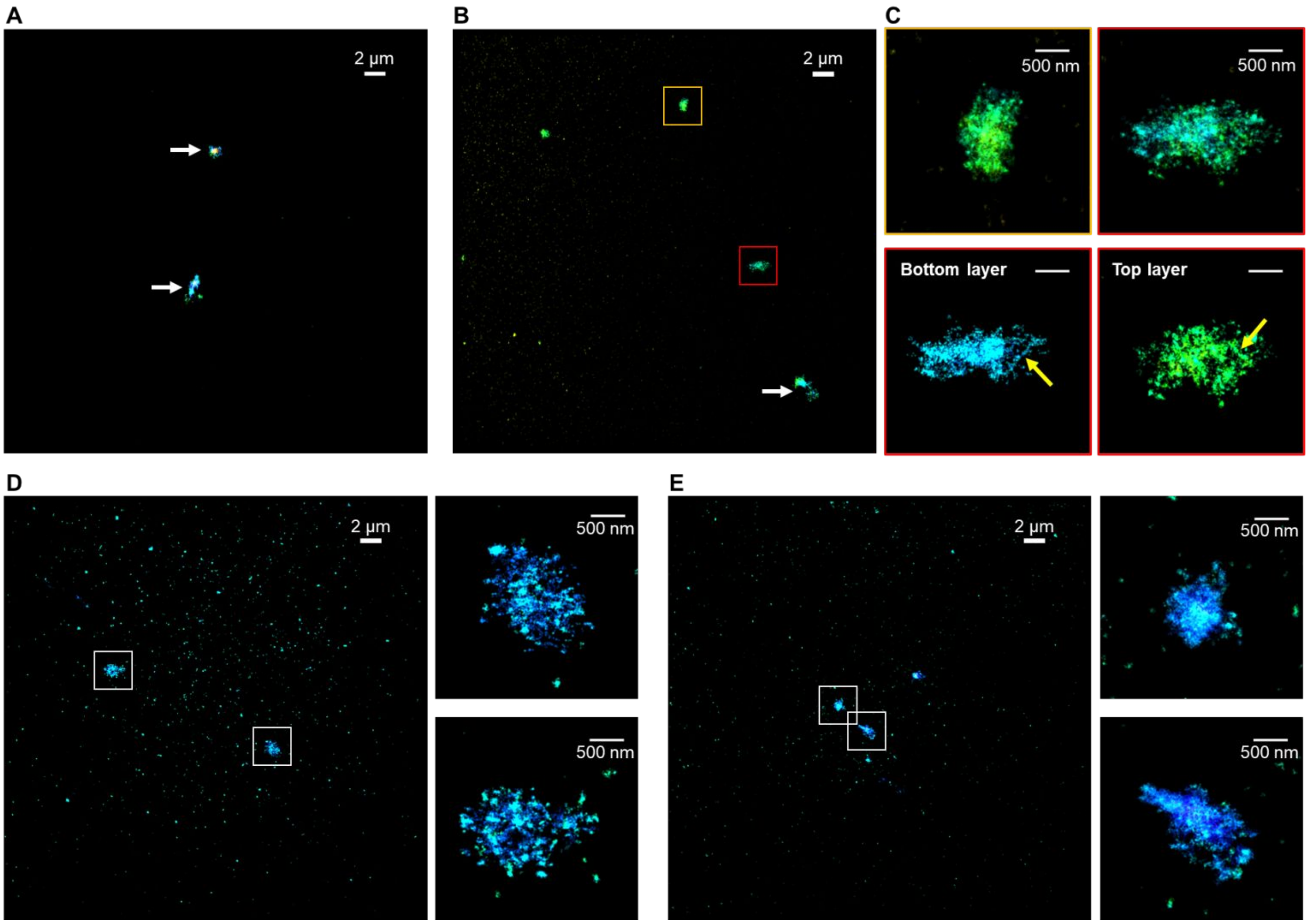

Figure S6. Additional 3D-STORM images of γPFD in *M. jannaschii*. (**A**) Zoom-out view of the 3D-STORM image containing the two cells shown in Figure 3B, 3D (arrows). Note that due to the extensive washing steps in our labeling protocol, we ended up with low cell counts in the final suspension, limiting the number of cells in the field of view. (**B**) Zoom-out view of another dataset. Arrow points to the cell shown in Figure 3E. (**C**) Zoom-ins for the two cells boxed in (B). The 3D-STORM data of the red-boxed cell is further separated by the depth Z into top and bottom layers, with yellow arrows pointing to filamentous structures. (**D,E**) Additional datasets with zoom-ins. Filamentous structures are generally observed, even though some cases are harder to resolve due to high γPFD density.

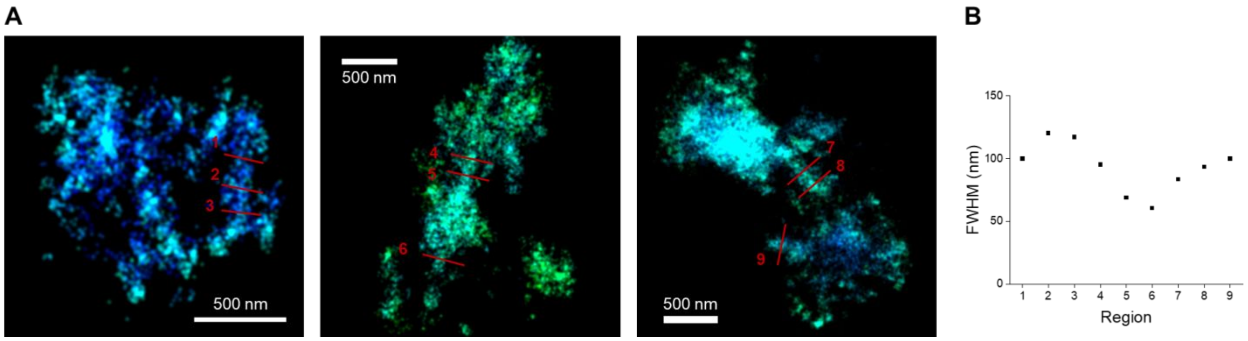

Figure S7. Additional width analysis for the filamentous structures of γPFD in *M. jannaschii*. (**A**) Enlarged figures of Figure 3C-E, with red lines marking where local cross sections are made. (**B**) FWHM of the different local cross sections.

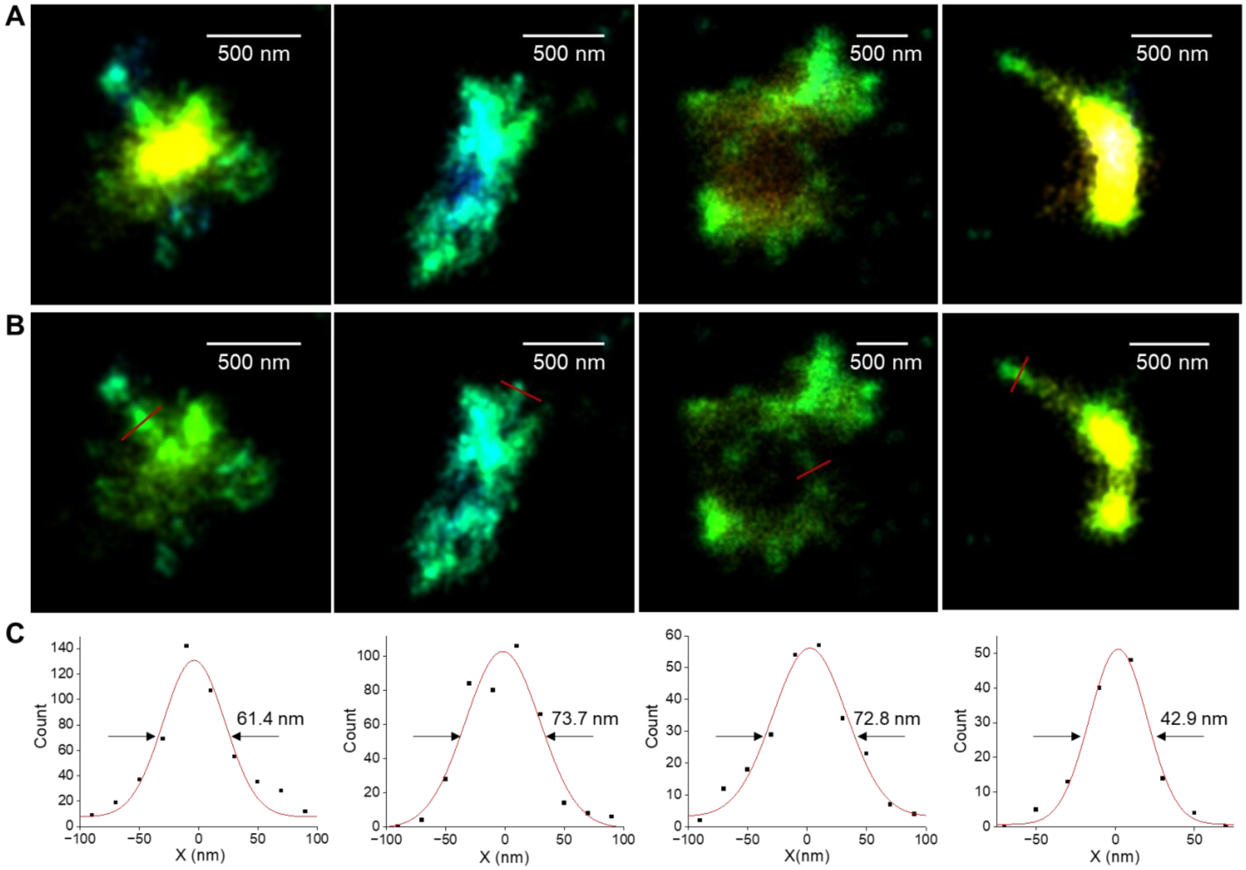

Figure S8. 3D-STORM of γPFD in *M. jannaschii* expressing His-tag γPFD and fixed with 3% paraformaldehyde plus 0.1% glutaraldehyde. The sample was treated with a freshly prepared 0.1% $NaBH_4$ solution and then immunolabeled using an anti-His-tag primary antibody and an AF647-tagged secondary antibody. (**A**) 3D-STORM images of 4 cells. (**B**) 400-nm thick Z-sections of the data in (A), to visualize the filamentous structures at the center of the cells. (**C**) STORM intensity profiles along the red lines marked in (B). Dots: experimental data; lines: Gaussian fits, with resultant FWHM values marked in each plot.

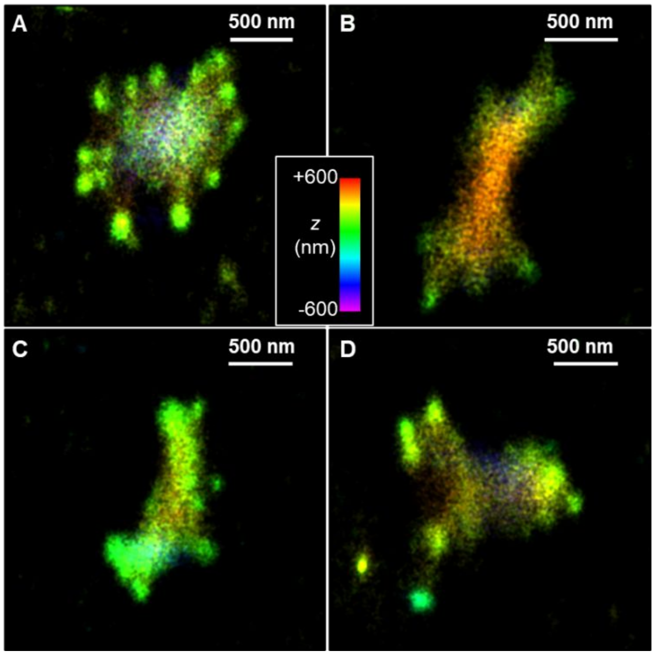

Figure S9. 3D-STORM of live *M. jannaschii* membrane (**A-D**) 3D-STORM images of live *jannaschii* membrane (color-coded for depth *Z* from -600 to +600 nm, with *Z* = 0 being the center of the focal plane) show round (**A**) and elongated (**B**-**D**) cell shapes.

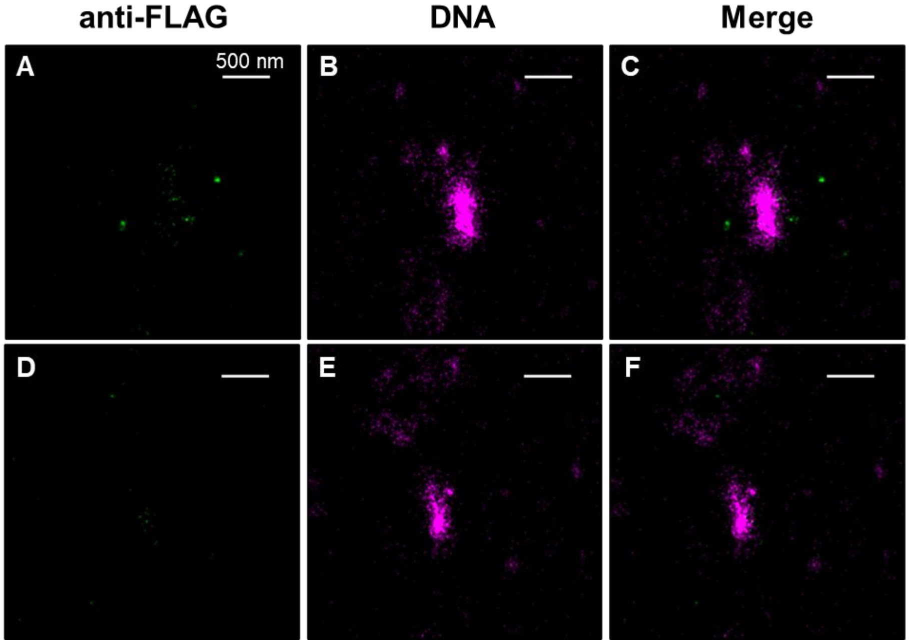

Figure S10. Two-color STORM imaging of control samples of wild-type *M. jannaschii* with no expression of 3xFLAG-γPFD. (**A**) and (**D**) STORM images in the 647-nm channel, showing no significant anti-FLAG signals. (**B**) and (**E**) PAINT of NucSpot Live 488-stained DNA in the 488-nm channel, showing structures comparable to that shown in Figure 4 for cells expressing 3xFLAG-γPFD. (**C**) and (**F**) overlaid images of the 647 and 488 channels.

References

(1) Glover, D. J.; Clark, D. S. Oligomeric assembly is required for chaperone activity of the filamentous γ-prefoldin. *The FEBS Journal* **2015**, *282* (15), 2985-2997.

(2) Winter, D. L.; Lebhar, H.; McCluskey, J. B.; Glover, D. J. A versatile multimodal chromatography strategy to rapidly purify protein nanostructures assembled in cell lysates. *Journal of Nanobiotechnology* **2023**, *21* (1), 1-15.

(3) Susanti, D.; Frazier, M. C.; Mukhopadhyay, B. A genetic system for *Methanocaldococcus jannaschii*: an evolutionary deeply rooted hyperthermophilic methanarchaeon. *Frontiers in microbiology* **2019**, *10*, 1256.

(4) Lim, S.; Jung, G. A.; Glover, D. J.; Clark, D. S. Enhanced Enzyme Activity through Scaffolding on Customizable Self-Assembling Protein Filaments. *Small* **2019**, *15* (20), 1805558.

(5) Lim, S. *Engineering Ultrastable Protein Scaffold for the Controlled Assembly of Multifunctional Nano-biomaterials*; University of California, Berkeley, 2019.

(6) Sun, Y.; Schöneberg, J.; Chen, X.; Jiang, T.; Kaplan, C.; Xu, K.; Pollard, T. D.; Drubin, D. G. Direct comparison of clathrin-mediated endocytosis in budding and fission yeast reveals conserved and evolvable features. *Elife* **2019**, *8*, e50749.

(7) Mund, M.; Kaplan, C.; Ries, J. Localization microscopy in yeast. In *Methods in Cell Biology*, Vol. 123; Elsevier, 2014; pp 253-271.

(8) Kaplan, C.; Ewers, H. Optimized sample preparation for single-molecule localization-based superresolution microscopy in yeast. *Nature protocols* **2015**, *10* (7), 1007-1021.

(9) Rines, D. R.; Thomann, D.; Dorn, J. F.; Goodwin, P.; Sorger, P. K. Live cell imaging of yeast. *Cold Spring Harb Protoc* **2011**, *2011* (9). DOI: 10.1101/pdb.top065482.

(10) Dempsey, G. T.; Vaughan, J. C.; Chen, K. H.; Bates, M.; Zhuang, X. Evaluation of fluorophores for optimal performance in localization-based super-resolution imaging. *Nature methods* **2011**, *8* (12), 1027-1036.

(11) Wojcik, M.; Hauser, M.; Li, W.; Moon, S.; Xu, K. Graphene-enabled electron microscopy and correlated super-resolution microscopy of wet cells. *Nature Communication* **2015**, *6* (1), 7384.

(12) Zhang, M.; Kenny, S. J.; Ge, L.; Xu, K.; Schekman, R. Translocation of interleukin-1β into a vesicle intermediate in autophagy-mediated secretion. *elife* **2015**, *4*, e11205.

(13) Huang, B.; Wang, W.; Bates, M.; Zhuang, X. Three-dimensional super-resolution imaging by stochastic optical reconstruction microscopy. *Science* **2008**, *319* (5864), 810-813.

(14) Rust, M. J.; Bates, M.; Zhuang, X. Sub-diffraction-limit imaging by stochastic optical reconstruction microscopy (STORM). *Nature methods* **2006**, *3* (10), 793-796.
